# Prognostic value of nutritional and functional screening instruments for mortality in patients with hematologic malignancies

**DOI:** 10.1007/s00520-026-10699-7

**Published:** 2026-05-01

**Authors:** Taise Andrade da Anunciação, Anna Karla Carneiro Roriz, Tícia Ranessa Santos Campos, Luana Milen Varjão, Catarina Lobo Santos de Souza, Ramona Souza da Silva Baqueiro Boulhosa, Pricilla de Almeida Moreira, Lilian Barbosa Ramos, Marco Aurélio Salvino de Araújo

**Affiliations:** 1 Postgraduate Program in Medicine and Health, Faculty of Medicine of Bahia, Federal University of Bahia, Salvador, Brazil; 2 Department of Nutrition Science, School of Nutrition, Federal University of Bahia, Salvador, Brazil; 3 Postgraduate Program in Food, Nutrition and Health, School of Nutrition, Federal University of Bahia, Salvador, Brazil; 4 Onco-Hematology Unit, Professor Edgard Santos University Hospital, Salvador, Bahia Brazil; 5 Midwest State University, Paraná, Guarapuava Brazil; 6 Department of Cardiovascular Science, College of Life Sciences, University of Leicester, University Road, Leicester, LE1 7RH UK

**Keywords:** Nutritional risk, Screening, Malnutrition, Survival, Mortality

## Abstract

**Purpose:**

Functional and nutritional impairments adversely affect outcomes in hematologic malignancies; however, the comparative prognostic value of widely available screening instruments remains insufficiently explored. The present study aimed to examine the association between nutritional and functional screening instruments and 12-month mortality in hospitalized patients with hematologic malignancies.

**Methods:**

In this prospective cohort of 91 patients (median age 53 years [IQR, 35–61]), nutritional screening and assessment tools (PG-SGA, GLIM criteria) and functional screening instruments (SARC-F, SARC-CalF, and Geriatric 8 [G8]) were applied within 72 h of admission. The G8 was pragmatically used as a multidimensional vulnerability screening instrument. Discriminative ability for mortality was evaluated using ROC curve analysis, and Cox regression models (unadjusted and adjusted) were used to examine associations with mortality.

**Results:**

During follow-up, 29.7% of patients died. The G8 and SARC-CalF were associated with mortality in adjusted models (HR 4.83, 95% CI 1.13–20.68; HR 2.37, 95% CI 1.08–5.17, respectively). PG-SGA Global showed acceptable discriminative performance in ROC analyses (AUC 0.71) but was not associated with mortality after adjustment. GLIM criteria were not associated with mortality in adjusted models.

**Conclusion:**

The G8 and SARC-CalF scores have demonstrated prognostic utility as simple and low-cost screening tools associated with mortality in these patients with hematological malignancies hospitalized in a resource-limited service.

**Supplementary Information:**

The online version contains supplementary material available at 10.1007/s00520-026-10699-7.

## Introduction

The clinical course of hematologic malignancies is frequently complicated by nutritional and functional impairment, which may compromise treatment response, prolong hospitalization, and increase mortality risk [1, 2]. Although malnutrition is consistently associated with worse clinical outcomes, there is still no universal consensus regarding diagnostic criteria, particularly in this population. Consequently, nutritional and functional evaluation has become an essential component of the management of patients with hematologic malignancies. However, comprehensive objective assessments may be difficult to implement in routine clinical practice, reinforcing the importance of rapid screening tools to identify patients at increased risk and prioritize further evaluation. The use of screening instruments prior to detailed nutritional assessment is therefore recommended [3].

Several instruments are available to identify nutritional risk. The Patient-Generated Subjective Global Assessment (PG-SGA) is widely used in oncology because it integrates symptoms, weight changes, and functional status, and is recommended by multiple consensus statements [3–5]. The Global Leadership Initiative on Malnutrition (GLIM) criteria provide an international framework for diagnosing and grading malnutrition [6, 7] and have demonstrated good sensitivity in hospitalized patients with cancer [8, 9]. In hematologic malignancies, the reported prevalence of malnutrition varies according to the instrument applied, ranging from 27.9% using PG-SGA [10] to 25.8% according to the GLIM criteria [1].

Beyond malnutrition, reductions in muscle mass and muscle function are highly prevalent in hematologic malignancies, with reported frequencies of low muscle mass ranging from 24 to 73%, depending on assessment methods and cutoff points [11]. Because muscle impairment is strongly associated with adverse clinical outcomes, current recommendations emphasize incorporating muscle-related parameters into routine nutritional and functional evaluation in this population [7, 12].

Rapid screening tools designed to capture functional and muscle-related vulnerability have therefore gained increasing attention. The Strength, Assistance with walking, Rise from a chair, Climb stairs, and Falls (SARC-F) questionnaire and its modified version incorporating calf circumference (SARC-CalF) are recommended for identifying risk of sarcopenia in clinical settings [13, 14]. The Geriatric 8 (G8) screening tool was originally developed to assess multidimensional vulnerability and has demonstrated prognostic value for survival and other adverse outcomes in patients with cancer [15].

Although the G8, SARC-F, and SARC-CalF were initially developed in older age groups, they assess fundamental domains of clinical vulnerability, including nutritional status, muscle strength, mobility, and functional decline. In oncology and hematology, these impairments are not restricted to older adults and may occur across the adult lifespan as a consequence of disease burden, systemic inflammation, and treatment-related toxicity [16, 17]. Accordingly, these instruments have increasingly been applied in adult cancer populations, supporting their pragmatic use beyond strictly geriatric contexts [18–22].

Despite their expanding clinical use, comparative evidence regarding the prognostic performance of nutritional and functional screening tools in hospitalized patients with hematologic malignancies remains limited. In particular, data on their ability to predict medium-term outcomes, such as 12-month mortality, are scarce [21, 23]. The availability of rapid, valid, and low-cost instruments is especially relevant in routine care and in resource-limited settings, where they may facilitate early risk stratification and support timely nutritional and functional interventions. However, the lack of robust comparative data continues to hinder broader clinical implementation and may contribute to the underrecognition of nutritional and functional impairment in this population.

Given these gaps, the present study aimed to examine the association between nutritional and functional screening instruments and 12-month mortality in hospitalized patients with hematologic malignancies, thereby clarifying their prognostic relevance and potential implications for clinical practice.

## Methods

A prospective observational cohort study was conducted between July 2021 and September 2022 in the oncohematology ward of the University Hospital in Brazil, after obtaining approval from the Institute Ethics Committee of the University Hospital (No. 4,439,123/2020). The study was conducted in accordance with the Declaration of Helsinki of 1975, revised in 2000. Written informed consent was obtained from all the patients before the nutrition screening.

### Sample

A consecutive sampling approach was adopted [24], including all patients admitted during the recruitment period who met the eligibility criteria. The sample size was defined based on the ward’s operational capacity, considering the average number of annual admissions (102/year), and the experience of previous studies conducted in similar populations at single centers [2, 21]. All included patients were followed for 12 months after admission to the study, with data collection on clinical outcomes, including mortality.

### Eligibility criteria

Patients aged 20 years or older, of both sexes, with a confirmed medical record diagnosis of a hematologic malignancy of multiple myeloma (MM), Hodgkin’s lymphoma (HL), non-Hodgkin’s lymphoma (NHL), acute myeloid leukemia (AML), or acute lymphoblastic leukemia (ALL), and able to participate in all stages of the study were included, with nutritional and functional data collection and follow-up.

Patients with a hospital stay of less than 24 h, cognitive limitations that hindered interview completion, hospitalization in the Intensive Care Unit with a reduced level of consciousness, receiving end-of-life care, or in respiratory and/or contact isolation were not eligible. Patients who did not participate in all stages of the study were subsequently excluded.

### Data collection

Patients were recruited during their stay in the ward and assessed within 72 h of admission using a semi-structured questionnaire supplemented with information from the electronic medical record. Covariates collected included age (adults < 60 years and older adults ≥ 60 years), sex, self-reported race/ethnicity (BAME (Black, Asian, and Minority Ethnic) and non-BAME categories), physical activity, presence of chronic comorbidities, number of medications, time since cancer diagnosis, history of antineoplastic treatment and hospital length of stay.

Data collection was performed by previously trained professionals, and a pilot project was conducted to test the feasibility of the collection methods and the operationalization of the survey. To ensure standardization of the assessment, each person was assessed by the same nutritionist.

### Nutritional and functional assessment

All patients underwent nutritional and functional assessment, including percentage of involuntary weight loss, Body Mass Index (BMI) [25, 26], and calf circumference [14], variables used in the screening instruments. The following validated instruments were used for nutritional and functional screening: PG-SGA, GLIM, SARC-F, SARC-CalF, and Geriatric 8 (G8).

### Patient-generated subjective global assessment (PG-SGA)

The PG-SGA instrument [27, 28] was used to assess nutritional status in patients diagnosed with cancer, assessing parameters such as weight loss, changes in dietary intake, gastrointestinal symptoms, functional capacity, and overall health perception. In this study, we used the Brazilian Portuguese version of the PG-SGA, translated and validated by Gonzalez et al. [28] and authorized by the PG-SGA/Pt-Global Platform (www.ptglobal.org).

In this study, we used the classification of the short form of the PG-SGA, considering the risk of malnutrition as low risk (0–3), medium risk (4–8), and high risk (> 9). The classification of the full version of the PG-SGA Global was used as follows: well-nourished (A), suspected of malnutrition or moderately malnourished (B), and severely malnourished (C) [27]. Subsequently, for statistical analysis, the overall PG-SGA classification was grouped into the following two categories: well-nourished (A) and at nutritional risk or malnourished (B + C).

### The global leadership initiative on malnutrition (GLIM) criteria

The diagnosis of malnutrition was assessed using the GLIM criteria [7], which combine phenotypic (involuntary weight loss, low body mass index, and reduced muscle mass) and etiologic parameters (reduced food intake or inflammation related to disease). Patients meeting at least one phenotypic and one etiologic criterion were classified as malnourished [6, 29].

Muscle mass reduction was assessed by calf circumference, with values < 34 cm for men and < 33 cm for women indicating muscle depletion [14].

Regarding the etiologic domain, both components recommended by the GLIM consensus were considered:

1. Reduced food intake, determined by patient report
2. Disease-related inflammation

Inflammation was assessed based on clinical judgment considering the underlying disease, clinical condition, and laboratory parameters, including C-reactive protein (CRP), in accordance with recent GLIM guidance [30].

In this cohort, all patients met at least one etiologic criterion, either due to reduced food intake and/or the presence of disease-related inflammation. Therefore, the differentiation of malnutrition status was primarily driven by the presence of phenotypic criteria.

Based on the combination of criteria, patients were classified as malnourished or well nourished according to the GLIM framework.

Strength, Assistance with walking, Rise from a chair, Climb stairs and Falls (SARC-F) and SARC-F + calf circumference (SARC-CalF).

The risk of sarcopenia was assessed using the Strength, Assistance with walking, Rise from a chair, Climb stairs, and Falls (SARC-F) questionnaire, as recommended by the European Working Group on Sarcopenia in Older People 2 (EWGSOP2) [13]. The instrument comprises five components evaluating difficulty with muscle strength, need for walking assistance, ability to rise from a chair, ability to climb stairs, and history of falls. The total score ranges from 0 to 10, with scores ≥ 4 indicating risk of sarcopenia [13].

In addition, sarcopenia risk was assessed using the SARC-F combined with calf circumference measurement (SARC-CalF), as proposed by Barbosa-Silva et al. [14]. This combined score ranges from 0 to 20, with scores ≥ 11 indicating risk of sarcopenia. Calf circumference was measured using an inextensible tape at the point of greatest horizontal protrusion of the calf. Reduced muscle mass was defined as calf circumference < 34 cm for men and < 33 cm for women [14, 21].

### Geriatric-8 (G8)

The Geriatric 8 (G8) instrument was used as a multidimensional vulnerability screening tool [15]. The instrument comprises eight domains, including food intake, weight loss, mobility, neuropsychological status, body mass index, medication use, self-perceived health status, and age. Participants were classified as having an abnormal score (G8 ≤ 14) or a normal score (G8 > 14) according to established cut-off values [16]. The established cut-off was applied pragmatically for exploratory purposes and does not represent formal validation of the instrument in younger adults.

### Outcome

The primary outcome was overall survival over 12 months, defined as the time between initial evaluation and death from any cause. Follow-up was conducted through the digital hospital information system and telephone contact. Survival data were also confirmed through the Local Death Registry Database. Time to death was calculated as the interval between the date of the first evaluation and the date of death. There were no missing data regarding patient survival.

### Statistical analysis

Continuous variables were expressed as means and standard deviations (SD) for normal distributions and as medians and interquartile ranges (IQRs) for non-normal distributions. Normality was verified using the Shapiro–Wilk test. Categorical variables were expressed as absolute frequencies and percentages. For comparisons between two independent groups, Student’s *t*-test (for normally distributed variables) or the Mann–Whitney test (for non-normally distributed variables) was used. Associations between categorical variables were assessed using Pearson’s chi-square test or, when appropriate, Fisher’s exact test (expected frequencies < 5).

Collinearity between independent variables was assessed using the Variance Inflation Factor (VIF) and tolerance values to avoid overfitting due to the limited number of events.

The predictive ability of screening instruments and clinical variables in relation to mortality was initially assessed using receiver operating characteristic (ROC) curve analysis for instruments providing graded or continuous scores, with accuracy expressed as the area under the curve (AUC) and corresponding 95% confidence intervals (95% CI). AUC values close to 1 indicate excellent discriminative ability, and values ≥ 0.75 were considered acceptable [31]. For receiver operating characteristic (ROC) curve analysis, the G8 score was inverted prior to curve estimation so that higher values of the transformed variable corresponded to a greater probability of mortality.

Indicators demonstrating adequate discriminative performance in ROC curve analysis were considered candidates for subsequent survival and multivariable Cox regression analyses. As the GLIM criteria represent a dichotomous diagnostic classification (malnutrition vs no malnutrition) rather than a graded or continuous risk score, ROC curve analysis was not applicable to this variable. Therefore, GLIM was directly evaluated in survival and Cox regression models to assess its prognostic association with mortality.

Survival was analyzed using the Kaplan–Meier method, with curves compared between groups using the log-rank test. Cox regression was used to identify variables associated with mortality, estimating hazard ratios (HR) and 95% confidence intervals (95% CI). Two models were constructed: Model 1 (unadjusted) and Model 2 (adjusted). Covariates included in the adjusted model were selected based on a combination of clinical relevance (age and sex) and results from preliminary bivariate analyses (*P* < 0.20).

The adjusted model included age (continuous) and sex (male/female), given their established clinical relevance, as well as the type of hematologic neoplasm (leukemia, lymphoma, or multiple myeloma) and presence of comorbidities (yes/no), based on their association in bivariate analyses. The type of hematologic malignancy was included in the model as a categorical variable with three categories (leukemia, lymphoma, and multiple myeloma) for adjustment purposes.

Nutritional and inflammatory variables such as BMI and CRP were not included as independent covariates due to potential collinearity with the instruments evaluated (G8, GLIM).

The proportional hazards assumption was assessed for all variables using both the Schoenfeld residual test and visual inspection of the scaled Schoenfeld residual plots. No violations of the proportionality assumption were identified (*P* > 0.05), indicating that the Cox model assumptions were adequately met. All statistical analyses were performed using Stata software, version 17.0 (StataCorp LLC, College Station, TX, USA), and graphs were constructed using GraphPad Prism software, version 8.0.1 (GraphPad Software, San Diego, CA, USA). The significance level was set at 5% (*P* < 0.05).

## Results

During the recruitment period, 129 patients were admitted to the ward, of whom 11 did not have a diagnosis of hematologic malignancy. Two patients declined to participate, eight did not meet the inclusion criteria due to physical or cognitive limitations, nine were in severe disease, five were in respiratory and/or contact isolation, and three did not complete all stages of the study. Therefore, the final cohort consisted of 91 patients diagnosed with hematologic malignancies.

The median age was 53 years (IQR, 35–61), with 71.4% classified as adults (< 60 years) and 58.2% were women. Most patients did not engage in regular physical activity (74.7%), and 58.2% had chronic comorbidities, primarily hypertension and type 2 diabetes mellitus. Among the patients, 36 (39.6%) had multiple myeloma, 33 (36.3%) had leukemia, and 22 (24.2%) had lymphoma.

During the 12-month follow-up, 27 patients (29.7%) died. Sociodemographic and clinical characteristics were comparable between patients who survived and those who died, except for the type of neoplasm. Leukemia was more frequent among patients who died compared to survivors (55.6% vs. 28.1%; *P* = 0.031) (Table 1).

**Table 1:** Sociodemographic, lifestyle, clinical, and nutritional characteristics by mortality status

Variables	Total (*n* = 91)	Mortality (29.67%)		*P*
		No (*n* = 64)	Yes (*n* = 27)	
	*n* (%)	*n* (%)	*n* (%)	
Hematologic malignancies				
*Leukemia*	33 (36.26)	18 (28.13)	15 (55.56)	**0.031**^**b**^
*Myeloma*	36 (39.56)	30 (46.88)	6 (22.22)	
*Lymphoma*	22 (24.18)	16 (25.0)	6 (22.22)	
Age, years, median (IQR)	53 (35–61)	51 (39–59.5)	55 (29–65)	0.528^a^
Age range				
Adults	65 (71.43)	48 (75.0)	17 (62.96)	0.246^b^
Older adults	26 (28.57)	16 (25.0)	10 (37.04)	
Sex				
Female	53 (58.24)	39 (60.94)	14 (51.85)	0.422^b^
Male	38 (41.76)	25 (39.06)	13 (48.15)	
Self-reported race/ethnicity				
No BAME	11 (12.09)	6 (9.38)	5 (18.52)	0.222^b^
BAME	80 (87.91)	58 (90.63)	22 (81.48)	
Physical activity				
No	68 (74.73)	48 (75.0)	20 (74.07)	0.926^b^
Yes	23 (25.27)	16 (25.0)	7 (25.93)	
Presence of chronic comorbidities				
No	38 (41.76)	30 (46.88)	8 (29.63)	0.128^b^
Yes	53 (58.24)	34 (53.13)	19 (70.37)	
Number of medications				
≤ 3	21 (23.08)	17 (26.56)	4 (14.81)	0.224^b^
≥ 4	70 (76.92)	47 (73.44)	23 (85.19)	
Time since diagnosis, months, median (IQR)	14 (4–28)	14 (3.5–23.5)	16 (4–33)	0.514^a^
History of antineoplastic treatment				
No	23 (25.27)	17 (26.56)	6 (22.22)	0.663^b^
Yes	68 (74.73)	47 (73.44)	21 (77.78)	
Hospital length of stay, days, median (IQR)	21 (11–26)	21 (13–25)	22 (10–30)	0.416^a^

*P*-value (*P*) with 5% significance (*P* < 0.05)

*IQR* interquartile ranges, *BAME* Black, Asian, and Minority Ethnic groups

^a^Mann–Whitney test

^b^Pearson’s chi-square

Regarding the nutritional and functional screening instruments, PG-SGA (Short) scores were higher among patients who died (median, 7.0; IQR, 2.0–13.0) than among survivors (median, 3.0; IQR, 1.0–6.0; *P* = 0.014). Similarly, scores from the full PG-SGA Global were higher among patients who died (median, 13.0; IQR, 5.0–18.0) compared with survivors (median, 5.5; IQR, 3.0–10.0; *P* = 0.001).

A higher proportion of severe malnutrition, according to the PG-SGA Global classification, is observed among patients who died (59.3%) compared with survivors (18.8%; *P* = 0.001) (Table 2). The SARC-CalF score was higher in the death group (median, 11.0; IQR, 6.0–14.0) than in survivors (median, 7.5; IQR, 1.0–11.5; *P* = 0.027). The G8 score was significantly lower among deaths (median, 10.0; IQR, 9.0–12.5) than among survivors (median, 13.0; IQR, 11.0–14.5; *P* < 0.001). Positive screening for multidimensional vulnerability using the G8 is more frequent among deaths (92.59%) than among survivors (68.75%; *P* = 0.016) (Table 2). No significant differences were observed between groups for the other instruments evaluated.

**Table 2:** Nutritional and functional screening instruments by mortality status

Variables	Mortality (29.67%)			
	Total (*n* = 91)	No (*n* = 64)	Yes (*n* = 27)	*P*
	*n* (%)	*n* (%)	*n* (%)	
Score PG-SGA (short), median (IQR)	3 (1–9)	3 (1–6)	7 (2–13)	**0.014**^**a**^
PG-SGA (short)				
Low risk	48 (52.75)	37 (57.81)	11 (40.74)	0.079^b^
Medium risk	18 (19.78)	14 (21.88)	4 (14.81)	
High risk	25 (27.47)	13 (20.31)	12 (44.44)	
Score PG-SGA global, median (IQR)	7 (3–13)	5.5 (3–10)	13 (5–18)	**0.001**^**a**^
PG-SGA global				
Well-nourished	17 (18.68)	14 (21.88)	3 (11.11)	
Suspected of malnutrition or moderately malnourished	46 (50.55)	38 (59.38)	8 (29.63)	**0.001**^**d**^
Severely malnourished	28 (30.77)	12 (18.75)	16 (59.26)	
GLIM				
Without malnutrition	32 (35.16)	26 (40.63)	6 (22.22)	0.093^c^
With malnutrition	59 (64.84)	38 (59.38)	21 (77.78)	
Score SARC-F, median (IQR)	2 (0–4)	1.5 (0–4)	2 (0–5)	0.445^a^
SARC-F				
No risk of sarcopenia	63 (69.23)	46 (71.88)	17 (62.96)	0.400^c^
Risk of sarcopenia	28 (30.77)	18 (28.13)	10 (37.04)	
Score SARC-CalF, median (IQR)	10 (1–12)	7.5 (1–11.5)	11 (6–14)	**0.027**^**a**^
SARC-CalF				
No risk of sarcopenia	56 (61.54)	43 (67.19)	13 (48.15)	0.088^c^
Risk of sarcopenia	35 (38.46)	21 (32.81)	14 (51.85)	
G8 screening	12.5 (10.5–14)	13 (11–14.5)	10 (9–12.5)	** < 0.001**^**a**^
G8				
Normal score	22 (24.18)	20 (31.25)	2 (7.41)	**0.015**^**d**^
Abnormal score	69 (75.82)	44 (68.75)	25 (92.59)	

*PG-SGA* patient-generated subjective global assessment, *GLIM* global leadership initiative on malnutrition, *SARC-F* strength, assistance with walking, rise from a chair, climb stairs and falls, *SARC-CalF* SARC-F + calf circumference, *G8* Geriatric 8, *IQR* interquartile ranges, *P P*-value with 5% significance (*P* < 0.05)

^a^Mann–Whitney test

^b^Pearson’s chi-square

^c^Student’s *t*-test

^d^Fisher’s exact test

**Fig. 1:**
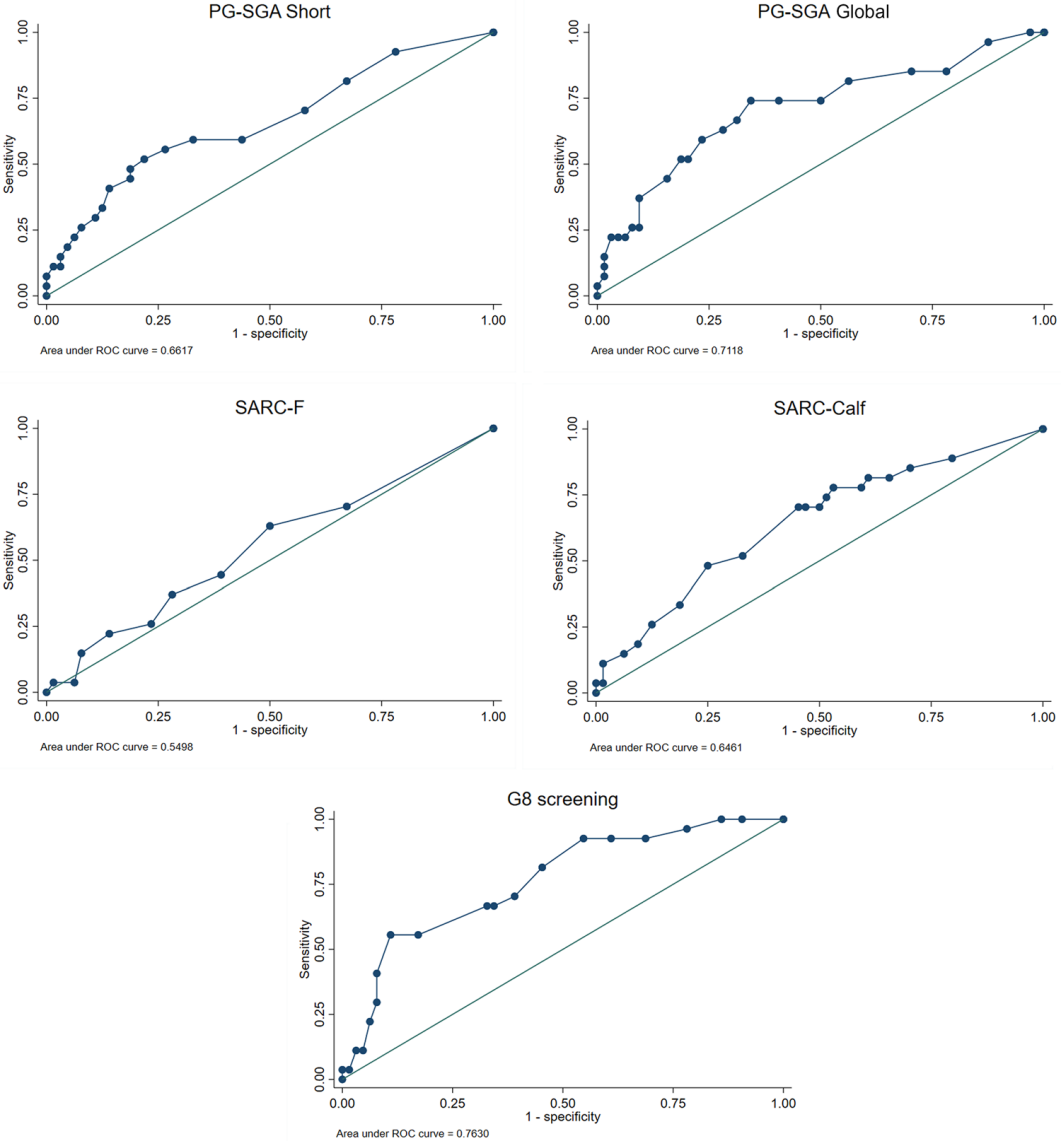
ROC curves and area under the curve (AUC) for screening instruments. PG-SGA, patient-generated subjective global assessment; SARC-F, strength, assistance with walking, rise from a chair, climb stairs and falls; SARC-CalF, SARC-F + calf circumference; G8, Geriatric 8. ROC, receiver operating characteristic; CI, confidence intervals

Figure 1 and Table 3 present the ROC curve analyses of the screening instruments for mortality discrimination. The G8 score demonstrated the highest discriminative performance, with an AUC of 0.763 (95% CI, 0.657–0.869), indicating acceptable accuracy. PG-SGA Global and SARC-CalF also showed moderate discriminative ability, with AUCs of 0.711 (95% CI, 0.588–0.835) and 0.646 (95% CI, 0.520–0.771), respectively.

**Table 3:** Discriminative ability of screening instruments for mortality

Variables	AUC	CI 95%	*P*
PG-SGA short	0.661	0.533–0.789	0.006
PG-SGA global	0.711	0.588–0.835	0.002
SARC-F	0.549	0.419–0.680	0.421
SARC-CalF	0.646	0.520–0.771	0.026
G8 screening	0.763	0.6571–0.86894	< 0.001

*PG-SGA* patient-generated subjective global assessment, *SARC-F* strength, assistance with walking, rise from a chair, climb stairs and falls, *SARC-CalF* SARC-F + calf circumference, *G8* Geriatric 8, *AUC* the area under the curve, *CI* confidence intervals, *P P*-value with 5% significance (*P* < 0.05)

In contrast, the SARC-F and PG-SGA short forms demonstrated lower discriminative performance and did not reach statistical significance.

Because the G8 is inversely scaled in relation to risk, lower scores correspond to a higher probability of mortality.

**Fig. 2:**
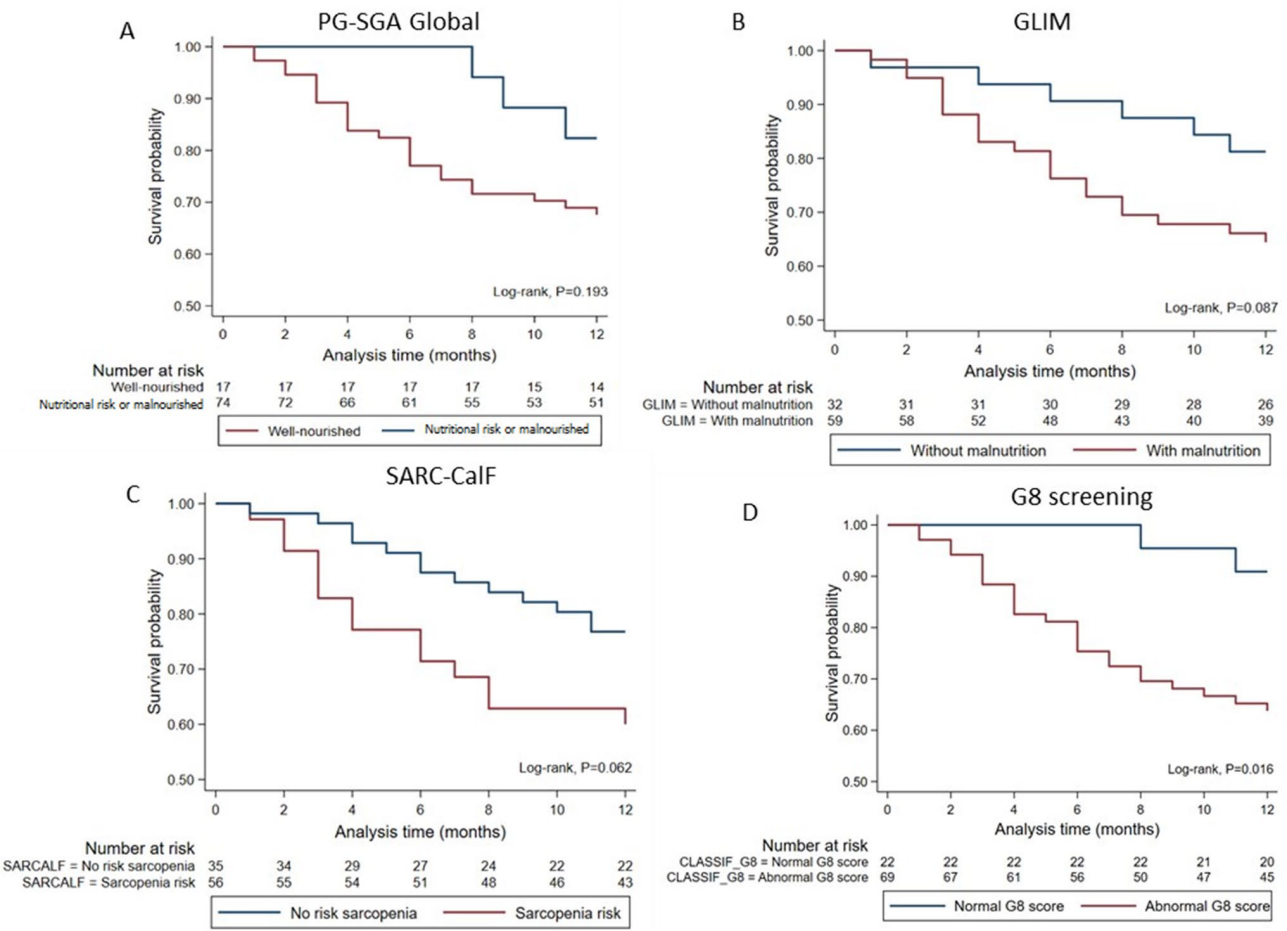
Kaplan–Meier survival curves according to screening instruments. PG-SGA, patient-generated subjective global assessment; GLIM, global leadership initiative on malnutrition; SARC-F + Calf, strength, assistance with walking, rise from a chair, climb stairs and falls + calf circumference; G8, Geriatric 8; log-rank test (*P* < 0.05).** A** PG-SGA, patient-generated subjective global assessment; **B** GLIM, global leadership initiative on malnutrition; **C **SARC-F + Calf, strength, assistance with walking, rise from a chair, climb stairs and falls + calf circumference; **D** G8,Geriatric 8

In the survival analysis, only the G8 screening shows a statistically significant association with 12-month mortality (Fig. 2). Patients with an altered G8 score (≤ 14) have a significantly lower 12-month survival probability compared with those with a normal score (> 14) (log-rank test, *P* = 0.016) (Fig. 2D).

**Fig. 3:**
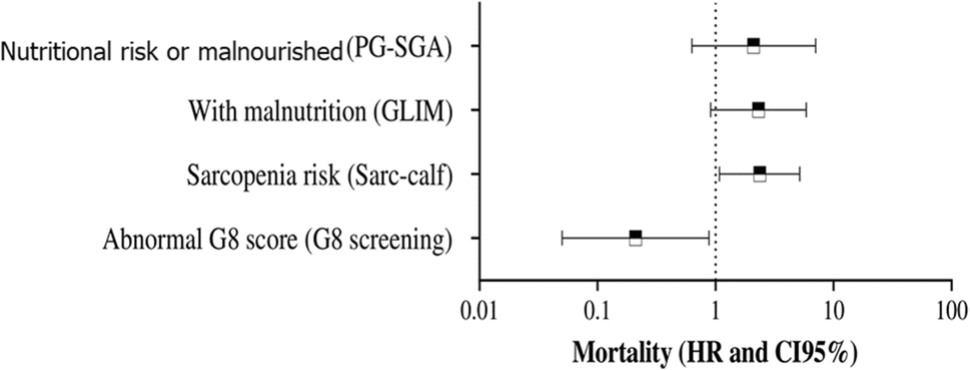
Cox regression analysis of screening instruments associated with mortality. PG-SGA, Patient-Generated Subjective Global Assessment; GLIM, Global Leadership Initiative on Malnutrition; SARC-F + Calf, strength, assistance with walking, rise from a chair, climb stairs and falls + calf circumference; G8, Geriatric 8; HR, hazard ratio; 95%CI, 95% confidence interval; *P*-value with 5% significance (*P* < 0.05)

In the Cox regression model adjusted for sex, age, comorbidities, and hematologic malignancy type (Fig. 3, Supplementary Table S2), sarcopenia risk identified by the SARC-CalF score remained significantly associated with increased mortality (HR, 2.37; 95% CI, 1.08–5.17; *P* = 0.031). Similarly, positive screening for multidimensional vulnerability using the G8 was associated with a higher risk of death (HR, 4.83; 95% CI, 1.13–20.68; *P* = 0.033), with lower G8 scores corresponding to greater mortality risk. It is important to note that, because the G8 operates on an inverse scale, lower G8 scores corresponded to higher mortality risk. In contrast, neither the PG-SGA Global score nor the GLIM criteria showed statistically significant associations with mortality in the adjusted models (*P* = 0.228 and *P* = 0.077, respectively).

## Discussion

This study is among the first to comparatively evaluate the prognostic performance of different nutritional and functional screening instruments applied during the same hospitalization episode in patients with hematologic malignancies, using 12-month mortality as the outcome. In adjusted analyses, G8 and SARC-CalF remained independently associated with mortality, reinforcing the relevance of early identification of nutritional and functional vulnerability in this clinical context.

Among the functional screening instruments, the G8 emerged as the only tool independently associated with survival in multivariable analyses. Patients with an abnormal G8 score (≤ 14) had an approximately 4.8-fold higher risk of death. As the G8 is inversely scaled, lower scores reflect greater vulnerability, which was consistently associated with higher mortality in our cohort. These findings suggest that the domains captured by the G8, including nutritional intake, weight loss, mobility, and functional decline, represent clinically meaningful markers of vulnerability in hospitalized patients with hematologic malignancies.

Previous studies have consistently demonstrated that abnormal G8 screening, indicative of multidimensional vulnerability, is associated with more advanced disease, poorer functional status, and reduced survival in patients with cancer [15, 32, 33]. This multidimensional vulnerability reflects diminished physiological reserve and increased susceptibility to treatment-related toxicity, infections, and functional decline [34–36]. Although the G8 was originally developed for older adults, the prognostic relevance of these vulnerability domains is not limited to chronological age and may be particularly pertinent in populations exposed to high inflammatory burden and intensive therapies, such as patients with hematologic malignancies [37–39]. In this context, the present study contributes by evaluating the prognostic performance of the G8 as a pragmatic screening instrument in a real-world inpatient hematology setting, where comprehensive geriatric or performance-based assessments are not routinely feasible.

The SARC-CalF, which combines the SARC-F questionnaire with calf circumference measurement, was also independently associated with mortality and outperformed the SARC-F alone. This finding highlights the added value of incorporating a simple anthropometric proxy of muscle mass into functional screening. Previous studies have demonstrated that the SARC-CalF correlates with handgrip strength, nutritional status assessed by the PG-SGA, and disease-related factors in patients with hematologic malignancies [21]. Given the high prevalence of muscle depletion and its prognostic implications in this population [11, 40, 41], the inclusion of calf circumference reinforces the importance of assessing muscle-related parameters, particularly when advanced body composition techniques are not available [29, 42].

Regarding nutritional screening, the full PG-SGA demonstrated discriminatory capacity in ROC analyses; however, its association with mortality did not remain statistically significant after multivariable adjustment. This finding may reflect the clinical profile of the cohort, which included a substantial proportion of patients with preserved body weight, relatively good functional status, and a limited burden of classic nutrition-impact symptoms at admission.

The PG-SGA remains a comprehensive instrument that integrates clinical history, physical examination, and metabolic stress, thereby providing a detailed evaluation of nutritional status [28, 43]. Nevertheless, its prognostic performance may vary according to population characteristics, disease severity, and clinical setting. Similarly, the GLIM criteria were not predictive of mortality in this cohort, possibly due to the low prevalence of key phenotypic criteria, such as significant weight loss or low body mass index. Although multicenter studies suggest that GLIM may be useful in certain subgroups, including lymphomas [44, 45], our findings indicate that its prognostic sensitivity in hospitalized patients with hematologic malignancies may be limited. Therefore, the absence of prognostic association observed in our cohort should be interpreted cautiously, and further studies with larger subgroup samples are needed to clarify potential differences across hematologic malignancies.

Overall, our results suggest that rapid, low-cost, and easy-to-administer screening tools applied early during hospitalization may help identify patients at higher risk of multidimensional vulnerability and, consequently, adverse outcomes, while supporting timely referral for comprehensive, multidisciplinary nutritional assessment. Rather than replacing structured evaluations, these instruments may function as an initial step within a tiered supportive care approach, particularly in resource-limited settings.

Some limitations should be acknowledged. First, nutritional and functional assessments were performed only at the beginning of the study; therefore, dynamic changes during hospitalization were not considered. Second, age was included as a covariate in the multivariate models, and the relatively small proportion of elderly patients limited the feasibility of age-stratified analyses.

Although the G8 and SARC-CalF were originally developed and validated in elderly populations, their components capture multidimensional domains of vulnerability, including reduced nutritional intake, weight loss, impaired mobility, and muscle function, which are not exclusive to chronological aging, as can be observed in studies in the field of oncology [16–22]. In patients with hematologic malignancies, these domains can be significantly influenced by disease burden, systemic inflammation, and treatment-related toxicity, regardless of age. Therefore, the use of these instruments in a predominantly non-elderly cohort aimed to explore whether such vulnerability constructs maintain prognostic relevance beyond the geriatric context. Consequently, this approach should be interpreted only as exploratory and does not constitute a validation study.

Thirdly, it was not possible to use the ECOG Performance Index (ECOG-PS), an established prognostic factor in oncology, because it was not part of the routine of the oncohematology team at the hospital in this research, which limited its analysis. The absence of ECOG-PS in this study may have resulted in residual confounding factors. Consequently, the observed associations between these screening tools and mortality should be interpreted with caution, as they may partially reflect underlying performance status not directly considered in the adjusted models.

Although patients receiving palliative care were excluded, residual confounding related to baseline functional status cannot be entirely ruled out. Future multicenter studies with larger and more heterogeneous populations are needed to validate these findings.

Thus, the prognostic associations observed for the G8 and SARC-CalF may reflect their ability to assess multidimensional vulnerability in hospitalized patients with hematologic malignancies, rather than an intention to replace established measures of oncological performance.

## Conclusions

In this prospective study, the G8 and SARC-CalF scores demonstrated prognostic utility as simple and low-cost screening tools associated with mortality in these patients with hematologic malignancies hospitalized in a resource-limited service. These findings highlight the relevance of integrating functional assessment and multidimensional vulnerability screening alongside nutritional evaluation during early inpatient measurement.

From an implementation standpoint, both instruments can be easily incorporated into hospital routine, as they are based on procedures accessible to clinical teams, including brief structured assessments and simple, low-cost anthropometric measurements.

Early identification of high-risk patients and this approach can help strengthen supportive care pathways in various healthcare settings, although confirmation in larger prospective studies is needed.

## Supplementary Information

Below is the link to the electronic supplementary material.

ESM 1 (DOC 49.5 KB)

## Data Availability

The authors have full control of the data and agree that the journal or reviewers may request access. Data are available from the corresponding author upon reasonable request.
