# Supplementary material for: Prognostic value of nutritional and functional screening instruments for mortality in patients with hematologic malignancies
Source: Support Care Cancer. 2026 May 1;34(5):484. doi: 10.1007/s00520-026-10699-7 (PMC13134974; doi:10.1007/s00520-026-10699-7)
Supplement: Supplementary file 1 — (DOC 49.5 KB) [file 520_2026_10699_MOESM1_ESM.doc]

**Supplementary Information**

Title of the article

Prognostic value of nutritional and functional screening instruments for mortality in patients with hematologic malignancies

Journal name

Supportive Care in Cancer

Authors

Taise Andrade da Anunciação¹, Anna Karla Carneiro Roriz², Tícia Ranessa Santos Campos³,

Luana Milen Varjão³, Catarina Lobo Santos de Souza⁴, Ramona Souza da Silva Baqueiro Boulhosa⁵,

Pricilla de Almeida Moreira⁶, Lilian Barbosa Ramos³, Marco Aurélio Salvino de Araújo¹

Affiliations

¹ Postgraduate Program in Medicine and Health, Faculty of Medicine of Bahia, Federal University of Bahia, Salvador, Brazil

² Department of Nutrition Science, School of Nutrition, Federal University of Bahia, Salvador, Brazil

³ Postgraduate Program in Food, Nutrition and Health, School of Nutrition, Federal University of Bahia, Salvador, Brazil

⁴ Onco-hematology Unit, Professor Edgar Santos University Hospital, Salvador, Brazil

⁵ Midwest State University, Paraná, Brazil

⁶ MRC Epidemiology Unit, University of Cambridge, Cambridge, United Kingdom

Corresponding author

Pricilla de Almeida Moreira

Research Study Assistant

MRC Epidemiology Unit, University of Cambridge

Email: pricilla.moreira@mrc-epid.cam.ac.uk

**Supplementary Table S1** Performance of nutritional and functional screening instruments in predicting mortality in individuals hospitalized with hematologic malignancy, based on the area under the curve (AUC) and 95% confidence intervals. Salvador, Brazil, 2025.

| **Variables** | **AUC** | **CI 95%** |
| --- | --- | --- |
| PG-SGA (Short) | 0.661 | 0.533 - 0.789 |
| PG-SGA Global | 0.711 | 0.588 - 0.835 |
| SARC-F | 0.549 | 0.419 - 0.680 |
| SARC-CalF | 0.646 | 0.520 - 0.771 |
| G8 | 0.763 | 0.6571 - 0.86894 |

PG-SGA, Patient-Generated Subjective Global Assessment; SARC-F, Strength, Assistance with walking, Rise from a chair, Climb stairsand Falls; SARC-CalF, SARC-F+calf circunference; G8, Geriatric 8. AUC, area under the curve; CI, confidence intervals.

| **Supplementary Table S2** Cox regression models for analyzing mortality in individuals hospitalized with hematologic malignancy according to nutritional and functional screening instruments. Salvador, Brazil, 2025. | | | | |
| --- | --- | --- | --- | --- |
| **Variables** | **Mortality** | | | |
|  | **Model 1** | | **Model 2** | |
|  | **HR (CI95%)** | **p** | **HR (CI95%)** | **p** |
| PG-SGA Global, *Malnutrition or malnourished* | 2.16 (0.65 - 7.16) | 0.210 | 2.10 (0.63 - 7.05) | 0.228 |
| GLIM, *with malnutrition* | 2.15 (0.87 - 5.32) | 0.099 | 2.31 (0.91 - 5.86) | 0.077 |
| SARC-CalF, *Sarcopenia risk* | 2.01 (0.94 - 4.27) | 0.071 | 2.37 (1.08 - 5.17) | **0.031** |
| Screening G8, *Abnormal* | 4.83 (1.14 - 20.43) | **0.032** | 4.83 (1.13 - 20.68) | **0.033** |

PG-SGA, Patient-Generated Subjective Global Assessment; GLIM, Global leadership initiative on malnutrition; SARC-F+Calf, Strength, Assistance with walking, Rise from a chair, Climb stairsand Falls+calf circunference; G8, Geriatric 8. HR, hazard ratio; 95%CI, 95% confidence interval; p-value with 5% significance (P<0.05).
